# Perspective-taking increases emotionality and empathy but does not reduce harmful biases against American Indians: Converging evidence from the museum and lab

**DOI:** 10.1371/journal.pone.0228784

**Published:** 2020-02-24

**Authors:** Aleksandra Sherman, Lani Cupo, Nancy Marie Mithlo

**Affiliations:** 1 Department of Cognitive Science, Occidental College, Los Angeles, California, United States of America; 2 Department of Gender Studies and Affiliated Faculty, American Indian Studies Center and Interdepartmental Program, University of California Los Angeles, Los Angeles, California, United States of America; Victoria University of Wellington, NEW ZEALAND

## Abstract

Given the problematic depictions of Native Americans and the pervasive cultural biases that exist, we sought to understand how contemporary educational practices in museums might encourage viewers to consider the context of their preconceptions rather than passively absorb conventional representations. In this two-part study, we tested whether and how viewers (mis)perceptions and interpretations of Native peoples might be influenced by encouraging empathy—specifically by taking the perspective of a Native individual depicted in a photograph they are visually analyzing. We randomly assigned participants in a lab setting (N = 120) and in a museum setting (N = 75) to one of three conditions (perspective-taking, stereotype-suppression, or control), and examined eye movements, self-reports, and verbal and written responses while participants viewed portrait photographs of American Indians. Notably, perspective-taking led viewers to interpret American Indians in a more emotional, empathetic, and human-centered manner than in control and suppression conditions. This was reflected in eye movements such that control and suppression participants attended to decorative features (e.g. jewelry) more than to the eyes of the depicted individual, whereas perspective-takers’ attention was more balanced. Similarly, perspective-takers used more empathetic and emotion-related language, whereas participants in control and suppression groups used more “objective” visually-descriptive language. Crucially, regardless of condition, cultural biases were stubbornly resistant to change and, in some cases, appeared even more frequently for participants adopting others’ perspectives. We argue that despite the positive outcomes associated with perspective-taking, the continued presence of cultural biases across conditions demonstrates that cultural competency-based interventions must be more complex and culturally-specific.

## Introduction

Research indicates that non-Indians possess little understanding of American Indian history and “have a foggy, distorted set of perceptions about Indians, usually based on little direct contact and what some admitted were little more than Hollywood stereotypes and generalizations” [1]. American Indian peoples in the U.S. are frequently depicted in dated and imaginative fictions that poorly reflect the lived realities of Native communities. Evidence of the ongoing diminishment of Native personhood and agency includes children’s literature and toys, dressing and playing Indian as entertainment or in commemoration of colonial desires, negative American Indian portrayals in film, the continued debate surrounding Native sports mascots (e.g. Washington Redskins), and derogatory mass-produced commercial goods. Art historians [2] state that “with few exceptions, the illustration of the Native American…[is]…an exercise of the imagination—or prejudice.” [2]. These problematic depictions of Native Americans and the pervasive cultural biases exist despite the continued efforts of educational programs to be progressive, inclusive, and multicultural. Thus, we asked how contemporary educational practices in museums might address these biased readings. What conceptual tools might be available to encourage viewers to consider the context of their preconceptions rather than passively absorb biased representations?

A multitude of psychological studies have shown that adopting others’ perspectives decreases stereotyping, increases positive attitudes, improves empathy, increases intergroup understanding, increases desire to engage in intergroup contact, and increases general social affiliation [3–6]. Additionally, research has demonstrated that perspective-takers rely less on egocentric judgments [7] and spontaneously seek out more information that is inconsistent with their expectations about others relative to control groups. This suggests that perspective-taking may undercut the default processing modes that lead to negative stereotyping [7]. Although the mechanisms by which perspective-taking does this aren’t completely clear, researchers propose that cognitive representations of the self and other merge during perspective-taking, whereby individuals see more of themselves in others, and more of others in themselves [8–10]. Moreover, the effects of perspective-taking may come about because it requires more complex, abstract, and deliberate thinking [11]. We thus investigated whether perspective-taking was a viable method for reducing biases against Native Americans.

In this two-part study, we expanded on prior research by investigating how perspective-taking shapes viewer’s perceptual, cognitive, and emotional responses to American Indian art and material culture. Specifically, we collaborated with the Autry Museum of the American West to assess whether museum visitors and Occidental College lab participants would perceive and interpret photographs of American Indians differently when encouraged to perspective-take. Our research was distinct from prior work in a number of ways. First, we systematically compared viewers’ interpretations of Native Americans in a controlled lab environment to a naturalistic museum environment. Additionally, whereas much work in social psychology tends to measure viewers’ biases by assessing how perspective-taking influences endorsement of stereotype-consistent (and inconsistent) statements, we aimed for a more naturalistic approach of assessing viewers’ spontaneous interpretations. Finally, we integrated eye tracking into our study to provide an implicit measure of how perspective-taking influences attention and gaze.

Crucially, we compared perspective-taking to two other conditions: stereotype-suppression and control. Suppression refers to an explicit attempt to suppress one’s own preconceived biases or biases that one knows to exist socio-culturally. Although this may be an intuitive strategy for expanding our current views, previous research demonstrates that suppression can rebound and lead to avoidance behaviors, causing individuals to ruminate on the biases they hope to eliminate, potentially causing further harm [6,12]. Based on prior research, we thus hypothesized that relative to control and suppression, a perspective-taking intervention would increase viewers’ empathy and positive attitudes towards Native Americans while decreasing bias, and that these effects would be reflected in eye gaze patterns, self-reports, and written/verbal descriptions. Specifically, we predicted that increased empathy would be reflected in increased attention directed towards the eyes compared to allocating attention toward objects and decorative qualities within the photograph (e.g. earrings, headpieces, clothing). We also expected that perspective-taking would lead participants to describe the depicted individuals in a more social, emotional, and human-centered manner relative to control and suppression. Our findings suggest that although perspective-taking can have positive effects, cultural biases about American Indians were stubbornly resistant to change and, in some cases, appeared even more frequently for participants encouraged to adopt others’ perspectives.

## Methods

All materials and procedures were approved by Occidental College’s Institutional Review Board. All participants signed informed consent prior to participation in the study. We report all measures, manipulations, and exclusions in these studies.

### Participants

Sample size was determined before data analysis. One hundred and twenty undergraduates (age range: 18–22, M = 21, 70 females; normal or corrected-to-normal vision) gave informed consent to participate for partial course credit. An equal number of participants were randomly assigned to each of the three between-subjects conditions (control *N* = 40; perspective-taking *N* = 40, suppression *N* = 40). Data from four lab participants was excluded from the analysis (1 due to error in photo presentation, and 3 due to demand characteristics—specifically they participated in the experiment the day after an election). Behavioral analyses thus included 40 control participants, 38 perspective-taking participants, and 38 suppression participants. An additional five participants were excluded from the eye-tracking analyses because their eyes were unable to be tracked for a significant portion of the trials leading to significantly fewer average fixations than other participants. The final eye-tracking analysis thus included 39 control participants, 36 perspective-taking participants, and 36 suppression participants.

Seventy-five museum visitors were recruited inside the Autry Museum of the American West museum either at the entrance or within the galleries. An equal number of participants were randomly assigned to each of the three between-subject’s conditions as above (control *N* = 25; perspective-taking *N* = 25, suppression *N* = 25). Data was collected and recorded using several iPads. Data from one iPad was corrupted (N = 16) and is therefore not included in analysis. The final museum analysis included 18 control participants, 23 in perspective-taking and 18 in suppression (age range: 19–79; M = 56, 34 females). Note that most museum visitors were older than our undergraduate population. This is related to the average age of museum goers nationally and was also related to the fact that although the Autry Museum of the American West does serve a high number of school children, they were not eligible to participate in this study. Sensitivity analyses were conducted using G*Power [13] for our key comparisons and are described in Results.

### Materials

**Fig 1:**
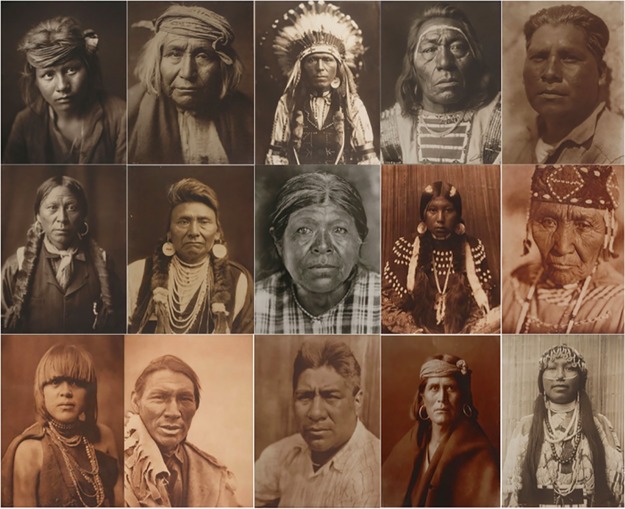
Final set of photographs chosen for inclusion in our lab portion of the study. Edward S. Curtis photos held by the Autry Museum of the American West. Top row (L to R): *A Son of the Desert—Navaho*, 1904 (Cur.4), *De Gizzeh—Apache*, 1906 (CUR.26), *Red Thunder—Nez Perce*, 1903 (CUR.36), *No Title*, early 1900s (CUR.18), *A Southern Dieguenño*, 1924 (CUR.234). Middle row (L to R): *No title*, 1904 (CUR.250), *Chief Joseph—Nez Perceé*, 1903 (CUR.258), *A Chukchansi Matron*, 1924 (P.37590), *Dusty Dress-Kalispel*, 1910 (CUR.1337), *Wife of Modoc Henry-Klamath*, 1923 (CUR.1545). Bottom row (L to R): *Povi-Tamu ("Flower Morning") -San Ildefonso*, 1925 (CUR.1681), *A Cree*, 1926 (CUR.1726), *Sam Ewing -Yurok*, 1923 (CUR.1537), *A Walpi Man*, 1921 (CUR.262), *Wishham Girl*, 1910 (CUR.40). Photos reprinted under a CC BY license, with permission from Autry Museum of the American West.

**Fig 2:**
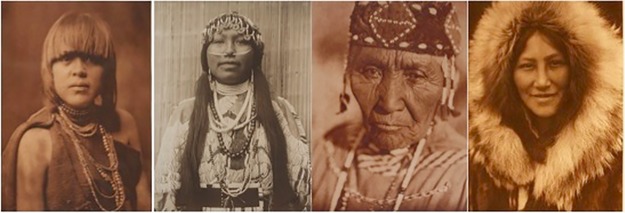
Edward Curtis photographs installed at the Autry Museum of the American West (February—May 2017). From L to R: *Povi-Tamu ("Flower Morning") -San Ildefonso*, 1925 (CUR.1681), *Wishham Girl*, 1910 (CUR.40), *Wife of Modoc Henry-Klamath*, 1923 (CUR.1545), *Ola—Noatak*, 1928 (2003.102.1.33). Photos reprinted under a CC BY license, with permission from Autry Museum of the American West.

In the lab, fifteen photographs of Native Americans taken by Edward S. Curtis between 1903–1928 were presented to participants in high resolution (1920 x 1080) on a computer (Fig 1). At the Autry museum, four Curtis photographs (Fig 2) were installed in a temporary exhibition space as components of a larger exhibition ("Art of the West") for a period of ten weeks (February 26-May 7, 2017). Three of these museum prompt photos were drawn from the lab selection of fifteen images and one was unique to the museum setting only.

We were particularly interested in working with portraits as we believed highlighting an individual’s face would focus the viewer on the individual personhood of each depicted individual and increase empathy overall. We also chose photographs of individuals with relatively neutral and/or ambiguous facial expressions. This ambiguity meant that participants’ mindsets could exert more robust effects on their interpretations. The final set of photographs included individuals who varied in gender, age, and the type of clothing they wore. Notably, to ensure that composition and other formal features did not strongly impact viewing and analysis, we chose photographs that were similarly sized (16” X 12”), similarly shot, and similarly colored.

### Lab procedures

Participants were told they would be viewing art photographs of Native Americans from the Autry Museum of the American West, that they would be asked to provide a series of responses after viewing each photograph, and that their gaze would be recorded via an eye tracker. In the lab, eye movements were monitored using an Eye Tribe eye tracker (resolution 0.1° [RMS] and a sampling rate of 30 Hz). Viewing distance (26cm) and head position were maintained using a chin rest. The height of the chin rest was adjusted for each participant so that participants’ eyes aligned with the center of the screen. A nine-point calibration was completed for each participant and continued until an accuracy of at least 0.30° of horizontal and vertical visual angle was achieved. Eye movements were recorded using pygaze [14] and stimulus presentation was controlled using OpenSesame [15]. The study was conducted in a dimly lit room.

To assess the extent to which mindset influenced how participants viewed and analyzed photographs of Native Americans, we randomly assigned participants to one of three between-subject conditions: perspective-taking, suppression, and control. Depending on the condition assignment, participants were provided with a set of instructions detailing how they should engage with the set of photographs to be presented. For control, participants were not given a specific set of viewing instructions. For perspective-taking, participants were instructed with the following prompt:

“As you view and engage with each photograph, please try to take the perspective of the individuals pictured. Imagine a day in their lives. Picture yourself living in their world and walking around in their shoes.”

For suppression, participants were instructed with the following prompt:

“Previous research has noted that our impressions and evaluations of others are consistently biased by stereotypic preconceptions. When viewing these photographs of Native individuals, please actively try to avoid thinking about the photographed individual in a stereotyped manner.”

Each trial began with a central fixation point and continued only after the participant remained fixated for three seconds. One of the fifteen photographs, presented in randomized order, was then displayed on the screen for eight seconds. Following each photograph, participants were asked to provide two sets of responses. First, participants were asked to use the paper provided to write a brief passage describing the photograph they just saw and any impressions and reactions they had to the photograph. Next, they were asked to rate how emotionally moved they were by the photograph using a 1–6 scale (1 indicated they were not at all emotionally moved, and 6 indicated they were extremely moved). Participants were encouraged to take their head off the chin rest during written responses and required to place their head back on the chin rest before beginning the next trial. At the end of the study, participants provided their age, gender and indicated if they had any experience with a Native community. Finally, participants were thanked and debriefed about the purpose of the study.

### Museum procedures

Museum visitors viewed each of four exhibited photographs in the same order for one minute. While viewing each photograph, participants’ physiological responses (e.g. heart rate variability) were measured using an Empatica wristband. Physiological data was not analyzed and is not reported in this manuscript. After viewing each photograph, participants were asked to verbally describe the photograph as well as any impressions and reactions they had to the photograph. These observations were recorded using an iPad and were later transcribed. After describing their impressions and reactions, participants verbally rated how emotionally moved they were using a 1–6 scale (1 indicated they were not at all emotionally moved, and 6 indicated they were extremely moved). After participants viewed all four images, they provided their age, gender and indicated if they had any experience with a Native community. Finally, participants were thanked, debriefed about the purpose of the study, and were asked whether they found the experience surprising or enriching.

## Results

### Eye movements

**Fig 3:**
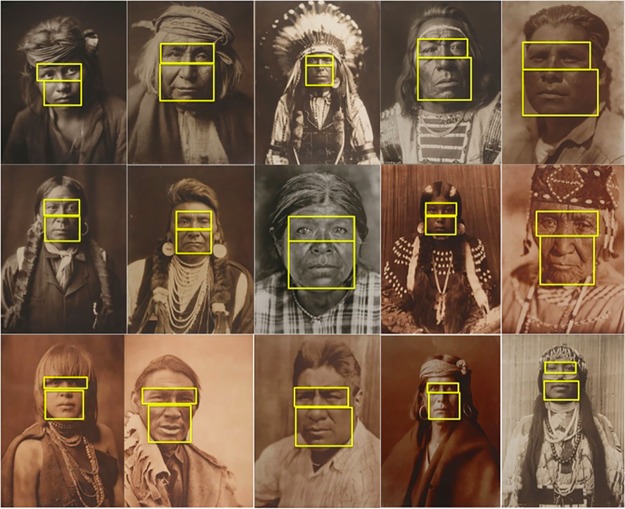
Predefined regions of interest (ROIs). Across participants, we compared fixations to the eye and face regions to fixations to object regions (anywhere outside of the yellow boxes).

We examined participants’ eye movements (lab setting only) to determine how attention was allocated and whether fixation locations differed depending on the condition (control, perspective-taking, suppression). We were particularly interested in comparing gaze allocations to the eyes with gaze allocations towards more decorative features of the photographs (e.g. clothing, jewelry, hair, ornaments, or background). For each of the fifteen presented photographs, decorative regions were defined as anywhere on the photograph that did not include the eyes, nose, or mouth (see Fig 3). Fixations were computed from raw eye movement data files consisting of time and position values using EyeMMV toolbox’s two-step spatial dispersion threshold algorithm [16]. For each participant, we computed the average proportion of fixations that were located within the eye region and subtracted this from the average proportion of fixations that were located on decorative regions. We separately computed the minimum required effect sizes to detect differences in fixation allocation for each group (suppression, *d* = 0.460; control *d* = 0.480; perspective-taking, *d* = 0.480) as final sample size varied by condition (suppression N = 39, two-tailed t-test, α = 0.05, 1-β = 0.80; control and perspective-taking N = 36, two-tailed t-test, α = 0.05, 1-β = 0.80).

**Fig 4:**
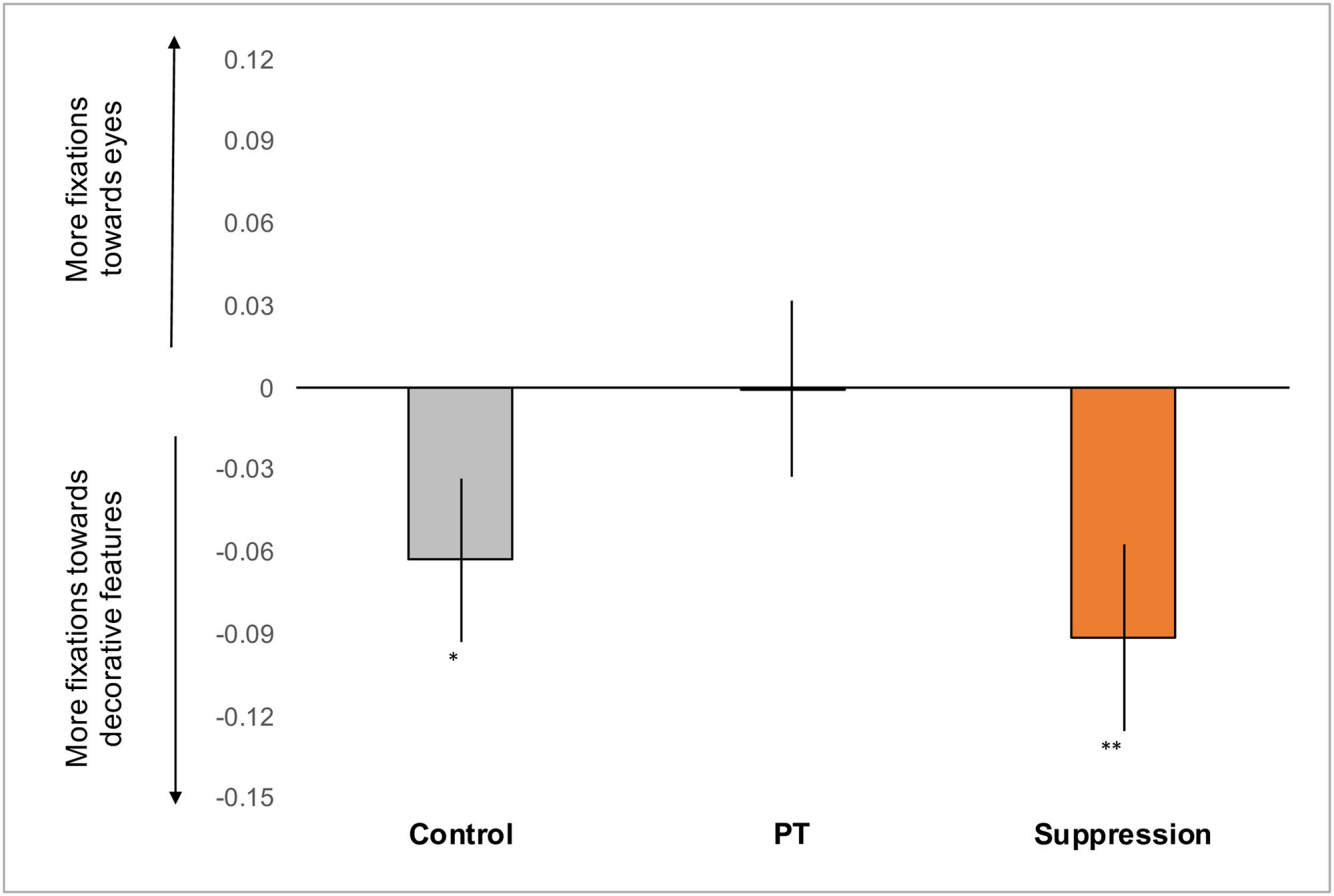
Average difference in proportion of fixations allocated to the eyes compared to the decorative features (average proportion of fixations in eyes minus average proportion of fixations in regions associated with decorative features). Error bars represent ±1 standard error of the mean (SEM).

**Fig 5:**
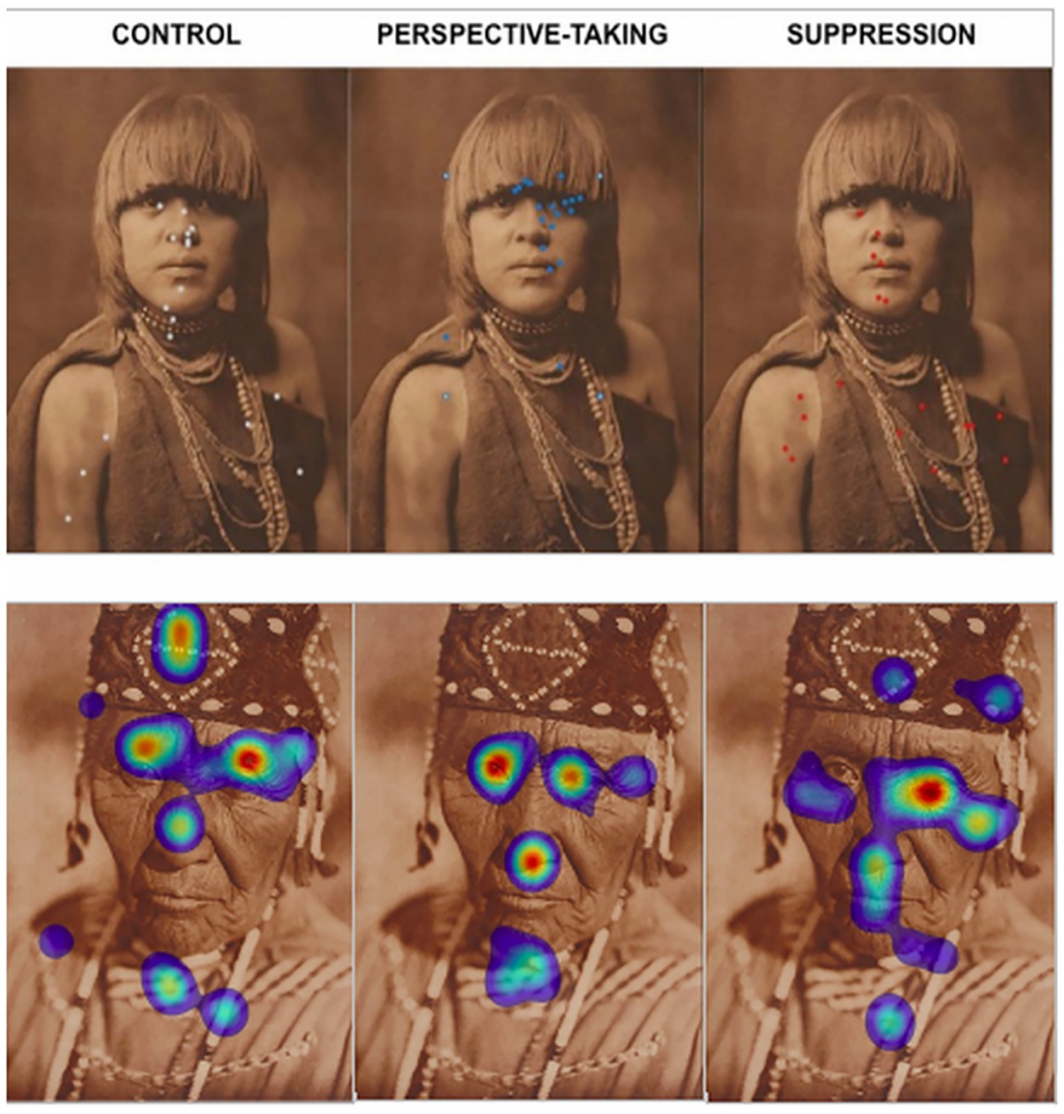
Distribution of gaze allocations (top) and a heat map (bottom) for a representative participant from each condition (left: Control, middle: Perspective-taking, right: Suppression).

As predicted, suppression caused participants to focus their attention toward decorative regions significantly more often than toward the eyes (*t*(38) = 2.660, *p* = 0.011, *d* = 0.443) (see Figs 4 and 5). Similarly, control participants directed attention towards decorative regions significantly more often than towards the eyes (*t*(35) = 2.122, *p* = 0.040, *d* = 0.339). In contrast, perspective-takers did not differ in the average proportion of fixations they allocated towards the eyes compared to decorative regions (*t*(35) = 0.008, *p* = 0.994, *d* = 0.001). Importantly, there were no significant differences in the average number of fixations between perspective-taking (*M* = 8.821, *SEM* = 0.159), control (*M* = 8.712, *SEM* = 0.189) and suppression (*M* = 8.878, *SEM* = 0.196), *F*(2,110) = 0.217, *p* = 0.805, η_p_^2^ = 0.004. Note that our observed effect sizes, though large, were slightly lower than determined by the sensitivity analyses suggesting that a larger sample would be needed to more effectively differentiate eye movements.

### Subjective reports of emotionality

Next, we examined how emotionally moved participants reported being in response to each photograph (self-reports ranging from 1–6). Sensitivity analyses conducted using G*Power (One-way ANOVA; N_lab_ = 116, α = 0.05, 1-β = 0.80; One-way ANOVA; N_museum_ = 59, groups = 3, α = 0.05, 1-β = 0.80) revealed the minimum required effect size to detect response-based (e.g. written or verbal responses, subjective reports of emotionality) differences between control, perspective-taking, and suppression in our lab (η_p_^2^ = 0.051), and museum samples (η_p_^2^ = 0.086). A separate sensitivity analysis (independent t-test; N_lab_ = 116, two-tailed α = 0.05, 1-β = 0.80) was conducted to compute the minimum required effect size to detect differences in responses between the lab and museum groups (*d* = 0.450).

We observed no significant differences between perspective-taking (*M* = 3.584, *SEM* = 0.113), control (*M* = 3.733, *SEM* = 0.119), or suppression (M = 3.624, *SEM* = 0.126) conditions for the lab context (*F*(2, 115) = 0.319, *p* = 0.728, η_p_^2^ = 0.005) and no differences between perspective-taking (*M* = 4.221, *SEM* = 0.169), control (M = 4.564, *SEM* = 0.195), or suppression (*M* = 4.139, *SEM* = 0.195) for museum participants, *F*(2, 58) = 2.139, *p* = 0.127, η_p_^2^ = 0.074. However, lab participants (*M* = 3.64, *SEM* = 0.07) reported being significantly less emotionally moved than did museum visitors (*M* = 4.32, *SEM* = 0.11), *t*(173) = 5.340, *p* < .0001, *d* = 0.864.

These findings are consistent with prior literature showing that engaging with real museum objects leads to more emotional investment than engaging with digital representations [17–21]. These results may also be explained by the demand characteristics associated with self-reports at a museum and with self-selection in attending a Native-focused art museum. Whereas in the lab, participants provided written responses, in the museum, participants provided experimenters with verbal responses. Additionally, because museum visitors were significantly older than lab participants, age effects cannot be ruled out.

### Impressions reflected in written and verbal descriptions

#### Word counts

We started our analysis of the qualitative responses by using a validated and reliable text-analysis software. The Linguistic Inquiry and Word Count (LIWC) text analysis software [22] allowed us to measure the number of words participants used that fell into specific categories of interest. The 2015 version of LIWC had built-in dictionaries to assess emotion-related words and we created custom dictionaries for the additional categories. Specifically, we created a custom dictionary to find the prevalence of words associated with stereotypes and conventional narratives. Whereas stereotypes tend to have negative connotations, the term conventional narrative is used to describe the tightly woven indicators of difference that may be positive as well [23]. For example, although words like “exotic” or “princess” do not have a negative valence, they demonstrate biased readings. Moreover, we were also interested in determining the prevalence of words indicating visual descriptions, words indicating cultural competency and sensitivity, empathy-related words, and words indicating curiosity and comfort with uncertainty. Table 1 details each word contained within our custom dictionaries. Differences across categories, conditions (control, perspective-taking, suppression), and contexts (lab, museum) were assessed using non-parametric independent samples Kruskal-Wallis tests.

**Table 1:** Words contained in each custom dictionary made for analysis using LIWC.

Conventional Narrative			Object Description				Eyes/Face		Cultural Competence	Empathy	Curiosity
Alcoholic	Hardlife	Rank	Accessor*	Fabric*	Paint	Stripe*	Cataract*	Line*	Appropriat*	Compassion*	Ambig*
Americanized	Hardship*	Ritual*	Adorn*	Fancy	Pant	Suede	Cloudy	Lip*	Assimil*	Connection	Curious*
Anglicized	Hardwork*	Rooted	Animal	Fashion	Pattern*	Suit	Empty	Mouth	Atrocit*	Empath*	Know*
Authentic	Heritage	Royal	Apparel	Feather	Piercing	T-shirt	Eye*	Nose	Capitalis*	Pity	Learn
Authority	Higher-status	Skinny	Attire	Flannel	Pigtail*	Tassl*	Foggy	Paint*	Coloniz*	Sorry	Relate
Battle	Horse	Socioeconomic	Bandana	Fringe	Pinstripe*	Trinket	Glanc*	Septum	Discriminat*	Sympath*	remind*
Beat	Humble	Spiritual	Bang*	Fur	Pixie	Tunic	Glar*	Skin	Displac*		Story
Been through a lot	Hung*	Status	Bead*	Garb	Plaid	Vest	Glass*	Wrinkl*	Euro*		Uncertain
Bride	Hunt*	Stem	Blanket	Garment*	Pocket	Wampum	Glaz*		Extinct		Unsure
Ceremon*	Ill*	Strage	Blazer	Garnet	Polka-dot*	Wear*	Glisten*		Fore*		Wonder*
Challeng*	Impoverished	Stress*	Bobcut	Hair*	Polo	Wrap*	Gloss*		Govern*		
Chief	Indigna*	Sweaty	Bone	Handkerchief	Poncho		Squint*		Integra*		
Clerk	Lead*	Tough	Bracelet*	Hat	Pony*		Star*		Mistreat*		
Cowboy	Malnourish*	Townsperson	Braid*	Head*	Quaffed		Tear*		Myth*		
Cultur*	Messy	Tradition*	Button*	Hoop*	Rag		Vacant		Oppress*		
Difficult	Modem	Tribal	Checker*	Jacket	Regalia		Watery		Persecut*		
Dirty	Money	Unclean	Cloth*	Jewel*	Ring		Ashy		Reductive		
Dusty	Musty	Underprivileged	Coat	Knot*	Scarf		Brow*		Stag*		
Econom*	Pocahantas	Untamed	Collar*	Leather	Shawl		Chapped				
Empower*	Poor	Warrior	Costume*	Material	Shirt		Cheek*				
Ethnic	Poverty	Weather*	Decora*	Metal	Shoulder		Chin				
Euro*	Power*	Wedding	Drap*	Multi-pattern	Shroud		Crack*				
Exoti*	Pretentious	West*	Dress*	Necklace	Simple		Crinkle*				
Farm*	Primitve	Work	Earring*	Nose-bone	Skins		Crow*				
Gambler	Princess	Weird	Elaborate	Ornate	Sleeve*		Fac*				
Grounded	Raqqed		Embellish*	Outfit	Slick*		Forehead				

Our primary interest was to assess whether participants’ visual analyses and interpretations reflected empathy and cultural sensitivity. Based on the prior literature, we predicted that participants in the control and suppression groups would be more “objective” in their responses, whereas participants in the perspective-taking condition would be more empathetic, would use more positive emotion words, would exhibit more cultural competence and curiosity, and would focus less on describing the objects in the photograph (jewelry, clothing) in favor of describing the individual.

**Fig 6:**
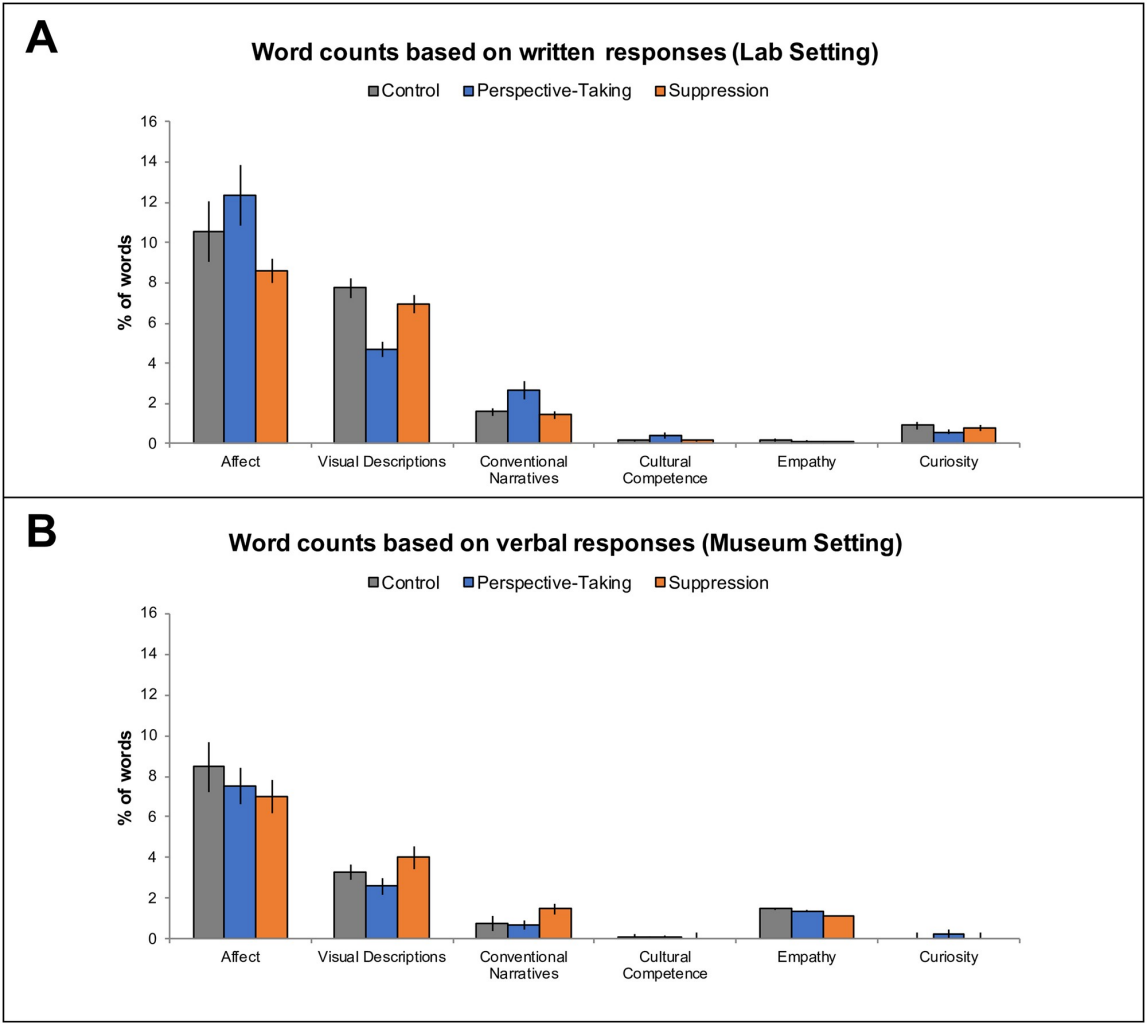
LIWC outputs (average percentage of words contained within each individual’s response) for A) lab and B) museum setting.

Our results were only partly consistent with our predictions (Fig 6A). In the lab setting, participants in the perspective-taking group (M = 12.320, SEM = 1.473) used significantly more emotion-related (both negative and positive) words than participants in control (M = 10.522, SEM = 1.522) and suppression (M = 8.582, SEM = 0.613), (*H*(2) = 6.097, *p* = 0.047, *ε*^2^ = .0005). However, in the museum, we did not observe differences in emotion-related word usage across conditions (*H*(2) = 1.545, *p* = 0.462, *ε*^2^ = 0.0001) (Fig 6B). Additionally, in the lab (*H*(2) = 7.313, = *p* = 0.026, *ε*^2^ = 0.0005), participants in the perspective-taking group (M = 4.697, SEM = 0.395) used significantly fewer visual descriptions than did participants in control (M = 7.737, SEM = 0.512) and suppression (M = 6.961, SEM = 0.439) but these differences were not significant in the museum (*H*(2) = 3.327, *p* = 0.189, *ε*^2^ = 0.0002).

Together with the eye-tracking results showing that participants assigned to control and suppression allocated attention towards the decorative features significantly more than towards the eyes, these findings may suggest that perspective-taking leads to increased empathy and deeper emotional connection relative to control and suppression. Contrastingly, control and suppression may lead viewers to, in a sense, “objectify” the depicted individuals; that is, whereas perspective-taking may be encouraging viewers to consider the personhood of the individual in the photograph, participants in control and suppression may feel more emotionally distanced from the individual and thus consider the visual features and objects more. A representative participant assigned to suppression exemplified this in their written response:

“*My attention went to the places of contrast*, *the white of her clothes and the reflection of her hair*. *I didn’t feel that emotional about this photo and I think that is because my attention was more on the details and less on the person*.*”*

One surprising finding is that there were no differences across condition in either the lab or museum in employing conventional narratives, or in exhibiting cultural competency, empathy, and curiosity (*H*(2)’s< 3.534, *p*’s > .171). In fact, based on this word count analysis, it would seem that participants employed very few conventional narratives, and at the same time exhibited little cultural competence, empathy, and curiosity. However, the LIWC outputs are limited by the fact that words are isolated and counted, rather than being considered in the context of a longer speech act. More complex and nuanced categories such as conventional narratives may thus appear significantly less often using a simplified word-counting approach. To further investigate whether conventional narratives were indeed employed, and whether the LIWC analysis underestimated the prevalence of these cultural biases due to the nature of the word-counting procedure, we conducted a more thorough coding-based analysis of our qualitative data.

#### Qualitative coding of written and verbal responses

To complement the LIWC results, three independent raters (co-authors) coded the qualitative data by determining whether any given response reflected one the following specific categories or thought processes: a) conventional narratives, b) visual descriptions, c) emotion-related judgments, d) self-related judgments e) curiosity and uncertainty, and f) historical assessments. After collation, for any ratings in which the coding was unreliable, the co-authors discussed the response (blind to condition and participant information) and agreed on a final rating leading to 100% agreement across categories. Note that all coders were blind to the conditions and all participant information. The coding criteria is outlined in Table 2. Each response was treated as an independent observation and subjected to chi-square goodness of fit tests. A sensitivity analysis (Goodness of fit *X*^*2*^ tests; Total observations = 1997, *df* = 2, α = 0.05, 1-β = 0.80; One-way ANOVA; N_museum_ = 59, α = 0.05, 1-β = 0.80) was conducted to compute the minimum required effect size (*φ* = 0.069) to detect differences between the lab and museum contexts for control, perspective-taking, and suppression in the percentage of verbal and written responses that fell into a particular category (e.g. conventional narrative).

**Table 2:** Qualitative coding criteria.

**Emotion-related Judgments**	Positive: Describe the subject positively (e.g.): “happy,” “proud,” “strong,” “powerful,” “wise,” “solemn knowledge,” “calm”. Negative: Describe the subject negatively (e.g.): “annoyed,” “bad temperament,” “strict,” “angry,” “not happy,” “twisted,” “miserable,” “tired”.
**Empathy**	Relates the subject to something/someone in their lives. Suggests they feel compassion and understanding for the person.
**Visual Descriptions**	Any reference to subjects’ attire, hairstyle, or headdress. “Objective” descriptions of what can explicitly be seen: Describing the quality of the photograph/coloring. Age, gender, physical features (e.g. eyes are glassy). Modern/traditional: but only insofar as it descriptive of what can be seen (modern clothing vs. traditional clothing).
**Conventional Narratives**	Employs narratives and biases they already have. May seem like descriptions but there is no evidence in photograph to support them. Seems to be jumping to a conclusion/creating a closed narrative. Creates fantastical or exoticizing narrative. Employs own standards to judge the person (“The jewelry or headdress they are wearing suggests they have a high status in society”) when statements are wrong. Seems to have small amount of knowledge that is applied inappropriately under the guise of the pan-Indian model (“I knew a Native person once,” “I just read a book on Natives,” “I just bought a pot in New Mexico,” “I always buy jewelry from Indians.”) Questioning the subject’s cultural authenticity based on their appearance or clothing. When two or more of the following stereotypical references are combined with closed narratives: Describing the subject as looking tired, dirty, lonely, isolated, worn out, exhausted, as having been through a lot, having survived so much, or as having “a tough past.” References to a subject looking proud, hard-working, resilient, or like a tribal leader.
**Curiosity/ Uncertainty**	Uses of the words “curious,” “questioning,” or “wonder.” Demonstrating that they want to know more about the subject’s story, not jumping to conclusions. Feeling enlightened/learned something new. Use of open-ended questions. Questioning whether the subject wants to be in the photograph or is forced into taking the photo.
**Historical Assessments**	Responses that include references to American imperialism, colonization, forced assimilation, or oppression. Responses to the time period specifically: “This is 1945…” Placing the photograph into history or questioning time frame (“I wonder when this was taken.”) Commenting on cultural norms.

The summary of responses is outlined in Table 3. Consistent with prior research, emotional judgments significantly varied by condition and context (*X*^*2*^(2) = 6.67, *p* = 0.035, *φ* = 0.073). Museum respondents used more emotion-related language (72%) than did lab participants (64%) and participants assigned to perspective-taking used more emotion-related language (73%) than did participants in the control (66%) or suppression groups (65%). Similarly, although there were few responses demonstrative of empathy overall (9%), museum visitors exhibited significantly more empathy (10%) than did lab participants (6%), and perspective-takers exhibited more empathy than control groups across both the lab and museum contexts (*X*^*2*^(2) = 12.27, *p* = 0.002, *φ* = 0.312).

**Table 3:** A summary of the percentage of responses by category, context (museum and lab), and condition (control, suppression, perspective-taking).

	Control		Perspective-taking		Suppression	
	Museum	Lab	Museum	Lab	Museum	Lab
Presence of emotion-related judgments	72%	60%	75%	72%	70%	59%
Exhibiting empathy	11%	5%	14%	7%	6%	5%
Presence of visual descriptions	56%	61%	45%	37%	53%	61%
Presence of conventional narratives	48%	40%	39%	60%	51%	7%
Exhibiting curiosity/uncertainty	14%	14%	23%	7%	7%	14%
Presence of historical assessment	19%	7%	13%	7%	8%	6%

Moreover, although the percentage of visual descriptions in the lab and museum contexts were similar overall (51% and 53%, respectively), participants assigned to control (59%) or suppression (57%) used significantly more visual descriptions than did perspective takers (41%) (*X*^*2*^(1) = 29.16, *p*< .0001, *φ* = 0.167). This result is in line with the eye tracking and LIWC results suggesting that perspective-takers may focus more on the person and their inner states, whereas participants assigned to control and suppression may focus more on visual descriptors such as objects.

With respect to conventional narratives, presence of curiosity, and presence of historical assessments, our results were only partly consistent with our predictions. Strikingly, about half of all respondents (46%) in both the lab and the museum expressed cultural bias by use of conventional narratives. Two representative samples below (one from the lab and one from the museum, respectively) indicate exoticizing and cultural fantasy:

“*He is very weathered*. *He seems to be connected with nature*. *He almost seems confused about why he is being photographed*. *I could see him as a dad who is stern but also loving*. *The chief in Pocahontas is my immediate first thought when I saw the image*. *I could see him riding a horse and being in battle*.*”*

(In response to CUR. 1726)

*“I think this is a really odd-looking image in a way*. *The hair looks butchered*. *It looks like it's almost super-added*, *wig-like*, *and so you have the child staring out from under this a little bit malevolently*. *Kind of a disturbing shadow across the top of the forehead there*, *so the child appears to be kind of looking out at us with a false pride*. *Then there's all the bling*, *you know*. *And that looks almost sort of classic dutch baby- the girl with the pearl earring*. *It’s this very odd juxtaposition and that image*. *But the total effect is kind of weird*.*”*

(In response to CUR.1681)

Particularly striking is the interaction between context and condition (*X*^*2*^(2) = 24.38, *p*< .0001, *φ* = 0.166). Specifically, although perspective-taking seemed to lead to fewer conventional narratives in the museum (39%) than control and suppression, lab participants who took the perspective of the depicted individual employed *more* conventional narratives (60%) than individuals in the control or suppression groups. Often, these conventional narratives were accompanied by a visual description suggesting that participants used the visual information to justify and confirm their biases. Example responses included:

*“[There is] A sense of yearning [in this individual]*. *Poor*, *sad*.*”*

(In response to CUR.4)

*“[This man] May not be friendly to get along with*. *Bad temperament*. *Strict*.*”*

(In response to CUR.281)

*“Old*, *poor*, *painstaking*, *experienced*, *she seems to have a hard life*.*”*

(In response to CUR.68)

*“I hold back my tears and anger as I remember someone I have lost*.*”*

(In response to CUR.218; *Note that here the respondent is narrating the perspective of the subject in the photograph)

Moreover, responses rarely featured open-ended questions and rarely indicated curiosity or comfort with ambiguity. Interestingly, although museum visitors assigned to perspective-taking seemed to express curiosity more often than the control groups, lab participants assigned to perspective taking exhibited less curiosity than control groups (*X*^*2*^(2) = 32.71, *p*< .0001, *φ* = 0.373).

Finally, few historical assessments appeared overall (11%) even in the museum where more historical context was present. Although viewers assumed the historic images were old and commented on the dichotomy between modern and contemporary, they rarely historically contextualized individual’s lived realities, which included warfare and genocidal political policies. If this recognition was present, the implications were minimized. A notable exception indicating historical assessment, empathy, curiosity, and cultural competence was:

*“Well*, *one was photographic*. *That wide open shutter so the face is exquisitely in focus and everything else is blurred out a bit*. *Also the timing*, *it being 1925 and a Modoc*, *this isn't that far after most her tribe would have been exterminated thanks you to the California Government*. *You can see all of the concern*, *the wisdom*, *the pain*, *all etched in that face*.*”*

(In response to CUR.1545)

Other participants did seem to both admit their lack of knowledge and asked questions. These open-ended and historically-framed responses should be a goal of future educational interventions.

*“Similar to the last image the woman seems expressionless and I believe the photograph had the intention of displaying her outfit and jewelry*. *My reaction is to wonder why she is wearing that outfit and what is she about to do*? *Or if she's about to do anything pertaining to that outfit at all*? *How does she feel being photographed in her Native look*?*”*

(In response to CUR.40)

## Discussion

Taken together, our data (including eye tracking, textual, and coding-based analyses) suggests a complex function of perspective-taking in mitigating cultural bias. Consistent with prior research, perspective-taking seems to lead viewers to interpret American Indians in a social, emotional, and human-centered manner. Specifically, whereas suppression and control participants were more likely to allocate their attention toward decorative features such as earrings or beadwork, perspective-taking led viewers to allocate attention in a more balanced way around the individual’s face. Moreover, perspective-taking caused participants to describe the individual using more emotional and empathetic language, and in some cases, to exhibit more curiosity than control groups; however, emotion-related judgments often exoticized the depicted individual. In the museum setting in particular, perspective-taking seemed to mitigate bias and increase curiosity relative to control groups. In contrast, in the lab setting, perspective-taking increased bias and decreased curiosity relative to control groups. Although it is possible perspective-taking is more effective in a real-world setting than in a lab environment in which participants may feel more distanced from the individuals depicted in the photographs, we are cautious to overinterpret these differences because of the demographic differences present across settings.

However, regardless of context and condition, participants interpretations still tended towards an unrealistic personhood that we believe reflects cultural bias. In fact, in the lab setting, perspective-taking seemed to *increase* the likelihood of employing conventional narratives. This set of findings appears to be in stark contrast with the research demonstrating positive impacts of perspective-taking on decreasing cultural biases. Below, we consider several ways to explain this seeming discrepancy, and provide recommendations for researchers and educators.

### Perspective-taking may be ineffective at mitigating cultural bias

While most researchers focus on the positive outcomes associated with perspective taking, several studies have revealed that although perspective-taking activates positive attitudes about others from an outgroup, stereotypes can continue to be maintained and unrevised. Particularly relevant is a phenomenon coined the “Ultimate Attribution Error,” which refers to one’s tendency to believe that negative outcomes are caused by an individual’s dispositions or traits when they are from an outgroup, but to believe that situational factors caused negative outcomes when individuals are from an ingroup. In contrast, positive outcomes are attributed to situational factors for outgroups, but to dispositions for members of an ingroup. Interestingly, perspective-taking lowers the likelihood of attribution errors. For example, research shows that after an African American male described social difficulties he experienced in college because of his race, participants who adopted his perspective attributed greater importance to situational causal factors, expressed more favorable attitudes toward African Americans in general, and reported feeling more empathy than did participants tasked with remaining objective and emotionally detached [24]. However, although perspective-taking increased situational attributions, it did not decrease dispositional attributions. This means that while participants increased their tendency to attribute the difficulties the black man experienced in college to situational factors (e.g. systemic racism), their tendency to attribute those difficulties to his disposition remained intact. Similarly, while perspective-taking led to an increase in pro-black attitudes in general, anti-black attitudes remained and did not decrease. This suggests that while perspective-taking can have positive impacts such as increased empathy and compassion, cultural biases can simultaneously remain entrenched and unrevised. This may explain why participants in our study may have experienced empathy while still endorsing conventional narratives.

Further support for the idea that perspective-taking can lead to positive attitudes about others from an out-group at the very same time as stereotypes are maintained comes from research on self-other merging. When self-other merging succeeds—that is, when one is able to perceive the other person as similar to oneself—perspective-taking often lead to positive outcomes. However, because perspective-taking is bidirectional in that individuals who put themselves “into another person’s shoes” ascribe positive characteristics about themselves towards others, *and* ascribe negative characteristics such as stereotypes about others onto themselves, perspective-taking can backfire when the perspective-taker has a negative self-view and believes that aspects of the self are threatened [24–26]. Additionally, when there seem to be either too large of differences between the self and other or unbridgeable differences between the self and other, perspective taking can also backfire. As Sassenrath et al. contend, “Unbridgeable gaps may be the result of well-learned differences (i.e., when taking the perspective of out-group members who are stereotyped as being very different from in-group members), but they may also result from single powerful experiences… Thus, although bridging the gap between the self and another person via perspective taking may generally produce positive social outcomes, some gaps are just too big and some people are just too different, making the task of perceiving the target as overlapping with the self almost impossible.” [25]. It’s important to note that in these cases, perspective-taking mechanisms are still activated, but do not produce positive outcomes. Although we do not have concrete evidence of this in our study, preliminary evidence for negative self-other merging comes from some participants assigned to perspective-taking who attempted to self-other merge so much so that they described the American Indian in the photograph using a first-person perspective (e.g. “I am neutral. My life has always had structure and I have never questioned it. Routine gives me purpose. I enjoy simple things, like animals and nature,”). These responses indicate that perspective-taking mechanisms and empathy are activated without positive outcomes, that both situational and dispositional attributions are made, and that biases and damaging judgments remain potentially because they are so entrenched and that the pictured individuals are perceived as too different from them.

Moreover, research suggests that the outcomes of perspective-taking or empathy based responses may be emotion-specific [27]. Particularly, the perceived emotion may be more or less challenging for the viewer to empathize with, as well as lead to differential outcomes. For example, viewers who perceived anger or disgust in the depicted individual may have struggled to empathize with, understand, or even contextualize these emotions without the proper knowledge base. Additionally, whereas feeling someone’s sadness may lead the viewer to adopt more prosocial attitudes and increase their helping behaviors, empathizing with someone else’s anger or disgust may lead to increased aggression [27]. In his recent book, *Against Empathy*, Paul Bloom similarly suggests that empathy is activated more for in-group than out-group members, and that feeling empathy is not enough to effect positive change. Rather, he suggests that compassion, which does not involve affective simulation, may be more important for mitigating biases [28].

These studies and our findings suggest that perspective-taking and empathy interventions may be overly broad and potentially inappropriate conceptual tools for researchers and educators to employ, especially for non-Native viewers who often have entrenched cultural biases toward American Indians. Psychologists [29] argue in their recent comprehensive study that “understanding the mind of another person is … enabled by *getting* perspective, not simply *taking* perspective.” [29]. They provide systematic empirical evidence showing that although perspective-taking has interpersonal benefits, it does not increase one’s ability to accurately understand the actual content another person’s mind. Only when participants have social knowledge available can perspective-taking enable them to make accurate inferences about others. When participants do not have the adequate knowledge or framework, they must gather new information in order to make accurate inferences rather than utilize their existing (and biased) knowledge of others.

Crucially, Eyal, Steffel, and Epley also show that their lack of findings supporting perspective-taking as an effective method for reducing stereotypes at the same time as increasing one’s cultural competencies, are not due to their perspective-taking manipulations lacking robustness. In their study, participants in perspective-taking conditions reported feeling confident that they were able to adopt another’s perspective and reported trying harder to do so than participants in the control conditions. Similarly, in our study, we don’t believe that the fact that perspective-taking failed to reduce cultural bias is the result of an ineffective manipulation on our part. We have no reason to believe that participants struggled to “put themselves into another person’s shoes,” and have evidence (e.g. several participants using first-person narration) that they feel they succeeded. Rather, we argue that a generalized method like perspective-taking while effective for increasing empathy and emotional investment, is simply not suited for decreasing conventional narratives. As we noted previously, the positive outcomes associated with perspective-taking may also be due to the specific dependent measures employed in previous studies (e.g. endorsement of stereotype-consistent statements).

### Limitations

There were several limitations to our study that may also explain the striking frequency with which participants employed conventional narratives. One potential source of this is the specific selection of photographs we chose. Curtis’ photographs are historic, which may have made our stimulus set less neutral than we anticipated and in turn led viewers to reify their preconceptions and biases about Natives as non-contemporaneous peoples.

Consistent with these claims, research has demonstrated that attempts to take another’s perspective may activate, rather than inhibit preconceptions and biases. People generally use social information they already have stored in memory to make rapid judgments, so it is unsurprising that the perceived stereotypicality of the person they are engaging with—that is, how strongly the other person seems, upon first glance, to fit into the stereotypical group—influences the degree to which conventional narratives are employed. If the subject of the gaze is not characteristically “different” from the viewer, the likelihood of endorsing biases during perspective-taking is decreased. Somewhat paradoxically, participants viewing a photograph of individual who is characteristically “other” either by looks or actions seems to activate more stereotypical assumptions even as the viewer attempts to take the perspective of that person [30]. Our choice to use historic photos may have thus made stereotypes more salient, resulting in viewers assigned to perspective-taking exhibiting more cultural biases than control groups who were not prompted to exercise empathy in looking.

Future research employing a more contemporary stimulus set (color photographs, contemporary dress, and/or the use of full-body photographs) may result in different findings. However, we emphasize that although we take these objections seriously, we are not convinced that the historic one-dimensional nature of Curtis’ photographs or the portrait convention of the images explains our findings. The popularity and prevalence of Curtis images of American Indians have rendered them typical and useful as generic images outside of chronological attributes. And, research has demonstrated time and time again that non-Natives perceptions and interpretations of contemporary Native Americans are riddled with cultural bias. Moreover, photos of contemporary American Indians may present an additional challenge in that visitors may reject that they are actually looking at a Native person. Therefore, while it is interesting and important to use a contemporary stimulus set, we predict that without the appropriate conceptual tools, conventional narratives will remain.

## Conclusions

Based on the evidence we provided both from prior research and through our data, we believe it will take a concerted effort that is more complex and nuanced to undercut cultural bias toward American Indians. To come to terms with our cultural biases, rather than employ generalized, one-dimensional interventions such as perspective-taking that may already rely on the individual having a strong cultural knowledge base, we suggest future research carefully consider more culturally-specific interventions. One potentially fruitful approach may be to encourage individuals viewing Native American art and material culture (and those interacting with Native peoples) to delay their interpretations and to reward uncertainty, curiosity, and comfort with ambiguity. Participants who were most curious and comfortable with “not knowing”, though few, seemed to tolerate difference more effectively, and to be less biased in general. The acceptance that one does not know a body of knowledge may not be an intuitive stance in an era where every answer may be found with the click of a button. Additionally, in a competitive commercial society, expression of certainty is a hallmark of self-possession and leadership. However, we believe that these traits are not productive in a learning environment with new and challenging material, and that uncertainty or forestalling closure in a learning encounter can enhance cultural competence. Lonnie G. Bunch III, the Director of Smithsonian’s National Museum of African American History and Culture Museum (NMAAHC) recently stated,

“What I want is a museum that helps the public embrace ambiguity. Because if you embrace the ambiguity, then it’s about the learning, it’s about realizing that there’s not one answer to anything. And to realize that complexity is the way to understand who you are today.”

[31]

This move to uncertainty also finds relevance in Indigenous Studies. Scholars at the University of New South Wales assert that decolonial goals cannot be enacted simply by confronting Western pedagogies. They speak of prioritizing learning dispositions that encourage openness and less certain positions because such thought requires more complex and nuanced argumentation and “prevent[s] slippage into forms of thinking and critical analysis that are confined within dichotomies between primitivism and modernity; and as a way to avoid the closed-mindedness of intellectual conformity” [32]. Thus, although perspective-taking may have some positive impacts such as increased empathy, future research should also consider how encouraging uncertainty may be an even more productive means to foster tolerance for difference.
